# Hyperandrogenism Does Not Influence Metabolic Parameters in Adolescent Girls with PCOS

**DOI:** 10.1155/2012/434830

**Published:** 2012-03-27

**Authors:** Kim Forrester-Dumont, Ovidiu Galescu, Andrey Kolesnikov, Nouhad Raissouni, Amrit Bhangoo, Svetlana Ten, Amy Suss

**Affiliations:** ^1^Division of Adolescent Medicine, The Children's Hospital at SUNY Downstate, Brooklyn, NY, USA; ^2^Pediatric Endocrinology Division, Infant's and Children's Hospital of Brooklyn at Maimonides and the Children's Hospital at SUNY Downstate, Brooklyn, NY 11219, USA

## Abstract

*Background.* Underlying insulin resistance and/or obesity has clearly been implicated in the development of metabolic syndrome in adolescents and young adults with polycystic ovarian syndrome (PCOS). It is not clear however what role hyperandrogenism has on the development of metabolic syndrome or its role on those metabolic parameters associated with metabolic syndrome. *Methods.* We studied 107 adolescent girls; 54 had PCOS according to NIH criteria. Data was obtained for systolic and diastolic blood pressure (SBP and DBP), body mass index (BMI), total testosterone (T), luteinizing hormone (LH), follicle-stimulating hormone (FSH), prolactin, fasting lipid profile, and glucose. The PCOS group was divided initially into subgroups according to BMI (kg/m^2^), then based on T (ng/dL) levels as follows: High Testosterone PCOS (HT), Intermediate Testosterone PCOS (IT), Obese and Normal Testosterone (ONT), and lean and normal T (Control, C). *t*-test analysis was performed in between all the groups. *Results.* There was no statistical difference between HT and IT, HT and ONT, or IT and ONT in SBP, DBP, fasting blood glucose, lipid panel, LH, FSH, and prolactin levels. The control group had lower SBP and BMI comparing with ONT, IT, and HT groups. There were no statistical differences found in DBP, fasting blood glucose, lipid panel, LH, FSH, or Prolactin. *Conclusion.* Metabolic profile in adolescent girls with PCOS is not affected by either the presence of hyperandrogenism or the degree of hyperandrogenism.

## 1. Introduction

Polycystic Ovarian Syndrome (PCOS) is a heterogeneous group of conditions that include anovulation, hyperandrogenism, and polycystic ovaries. PCOS is often associated with both obesity and metabolic syndrome.

 The NIH-National Institute of Child Health and Human Development Conference of PCOS originally recommended that the major criteria for PCOS should include hyperandrogenism and/or hyperandrogenemia, oligoovulation, ultrasound pattern of polycystic ovaries and the exclusion of other known disorders. A PCOS diagnosis is considered a hyperandrogenic disorder of exclusion with an ovarian etiology. In 2003, the Rotterdam consensus expanded the diagnostic criteria to include at least two of the following three features: clinical and/or biochemical hyperandrogenism, oligoanovulation, and polycystic ovaries, excluding any other endocrinopathies [1]. In 2006, the Androgen Excess-PCOS society recommended that PCOS be defined by clinical and/or biochemical hyperandrogenism, with either oligoanovulation and/or polycystic ovaries, excluding related disorders [2].

 The circulating total and free testosterone and dehydroepiandrosterone sulfate (DHEAS) levels are elevated in 50–75% of women with PCOS, if high quality assays are used [3]. Androgen excess is considered by some investigators to be the key feature of PCOS; however, only 80–85% of women with clinical hyperandrogenism have PCOS [4, 5]. Despite this evidence hyperandrogenism remains an important part of PCOS and is included as major set of symptoms in both NIH and Rotterdam criteria [1]. According to the NIH criteria PCOS affects 6–10% of women of the child bearing age [6–10]. As per the broader Rotterdam criteria even more individuals can be classified as having PCOS [11]. Therefore PCOS becomes one of the most common human disorders and the single most common endocrinopathy in women of reproductive age.

According to a common view PCOS is a multifactorial and polygenic in nature [1, 11, 12]. Up to now multiple studies failed to identify genes responsible for PCOS and condition [12].

Metabolic syndrome is associated with development of hyperandrogenism and PCOS. But the question still remains unanswered as to what is primary or secondary: metabolic syndrome or hyperandrogenism. There is an opinion and data suggests that testosterone was significantly related to metabolic syndrome and its components in obese adolescent girls [13, 14]. Other data reveals that in obese adolescent girls PCOS does not add additional risk of further complications of metabolic syndrome [15, 16]. PCOS adolescent girls have been found to have increased visceral abdominal fat [16, 17], but there was no correlation between visceral abdominal tissue and total testosterone or free androgen index [15, 16]. There are case studies that show that low HDL cholesterol is the criterion which best explains the high prevalence of the metabolic syndrome in PCOS subjects which, in turn, is influenced by hyperinsulinemia, rather than by hyperandrogenemia [18].

In both animal and human models the associations between serum testosterone and insulin resistance or metabolic syndrome/type 2 diabetes/PCOS risk in women were demonstrated, but their cross-sectional nature did not allow conclusions about causality [19].

As such the aim of our study was to find out whether elevated testosterone level impacts the presentation of metabolic syndrome in obese adolescent girls.

## 2. Materials and Methods

### 2.1. Patients

We enrolled 107 adolescent girls from the pediatric endocrinology clinics and adolescent clinic at SUNY Downstate Medical Center. PCOS group was selected based on NIH criteria including irregular menses and clinical and biochemical signs of hyperandrogenism. The study was approved by IRB of SUNY Downstate Medical Center.

### 2.2. Exclusion Criteria

Congenital Adrenal Hyperplasia, Cushing's Syndrome, severe insulin resistance, and androgen-secreting tumors were excluded in all cases. Also excluded from this study were patients under treatment with any lipid-lowering drugs, insulin sensitizers (e.g., Metformin), or Inositol supplements.

The control group was selected from the adolescent clinic patients seen for routine health examinations. The majority of the patients were of African American descent.

The patients were divided into 4 subgroups.

1. High Testosterone (HT): patients that met the NIH and Rotterdam Criteria for PCOS and had Total Testosterone > 90 ng/dL;
2. Intermediate Testosterone (IT): patients that met the NIH and Rotterdam Criteria for PCOS and had Total Testosterone in range 51–90 ng/dL;
3. Obese and Normal Testosterone (ONT): patients that did not fulfill the NIH and Rotterdam Criteria for PCOS and had BMI > 95th percentile, total testosterone < 50 ng/dL;
4. Controls (c) Patients that did not fulfill the NIH and Rotterdam Criteria for PCOS and had BMI < 95th percentile, Total Testosterone < 50 and regular periods, and did not meet any of the exclusion criteria.

The group of 54 girls who had PCOS according to NIH criteria including irregular period or amenorrhea and increased level of testosterone was subdivided into 2 subgroups with (a) 23 patients with high level of testosterone (T) and (b) 31 patients with intermediate level of testosterone (T).

As a control we assigned 15 patients who had normal BMI and normal level of testosterone.

The ONT group consisted of 38 patients who had elevated BMI and normal testosterone.

Characteristics of the patients are detailed in Table 1.

### 2.3. Measurements Methods

Data was obtained for blood pressure, BMI, testosterone, LH, FSH, prolactin, fasting lipid profile, and glucose. BMI was calculated as weight (kg)/(height (cm))^2^ and BMI above 95th percentile according to the CDC [20], BMI charts was defined as obese.

Testosterone was determined by chemiluminescent immunoassay methodology; high testosterone was defined as testosterone above 90 ng/dL and high intermediate as between 51 and 90 ng/dL.

### 2.4. Statistical Analyses

Two-sample *t*-test assuming unequal variances analysis was performed to compare the means of the studied parameter between the 4 groups.

Multivariate logistic regression analysis was done for testosterone levels and LH, FSH, prolactin, lipid profile, fasting glucose, SBP, DBP, and BMI. For the statistical analysis we used the SPSS 19.0 software.

## 3. Results

 There was no statistical difference between the high testosterone group HT and the other groups IT and ONT in SBP, DBP, fasting blood glucose, lipid panel, LH, FSH, and prolactin.

Furthermore our analyses did not yield any statistically significant difference between the intermediate testosterone group IT and ONT in any of the studied parameters (Table 1).

**Figure 1:**
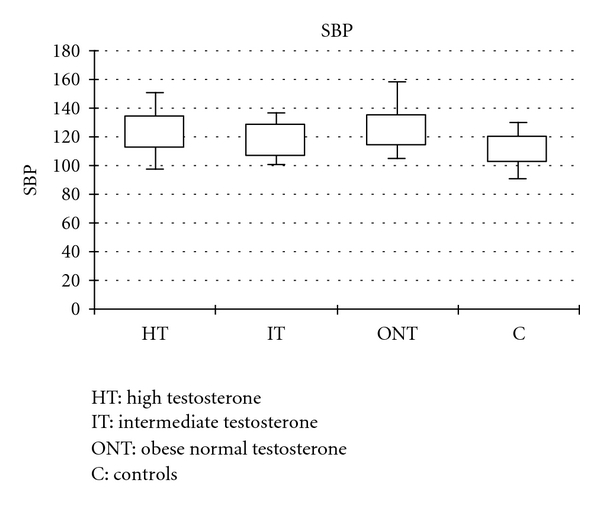
Systolic Blood Pressure—distribution among the groups. HT: High Testosterone, IT: Intermediate Testosterone; ONT: Obese Normal Testosterone; C: Controls.

The control group had lower SBP and BMI comparing with the other groups but no statistical difference was found in DBP, fasting blood glucose, lipid panel, LH, FSH, or prolactin (Figure 1). The regression analysis did not yield any correlation between the testosterone levels and any of the other parameters.

The regressions were run both for the entire population studied and for each separate subgroup.

BMI had statistically significant correlations with SBP, DBP, cholesterol profile, and LH but no correlation was seen for triglycerides, FSH, and prolactin (Table 2).

## 4. Discussion

 Upon review of the literature, several studies suggest that PCOS and high testosterone is an additional risk and is significantly related to metabolic syndrome and its sequelae in obese adolescent girls [21–25]. PCOS in adolescent patients has been found to have increased visceral abdominal fat [17], but there was no correlation between visceral abdominal tissue and total testosterone or free androgen index [15]. An elevated risk profile for cardiovascular morbidity was demonstrated in PCOS patients, mainly attributed to increases in type 2 diabetes, dyslipidemia, and hypertension [18, 26, 27] which might constitute a cofounder when studying the correlation between hyperandrogenism and the risk of metabolic syndrome in PCOS.

 However, contrary with such studies [28, 29], we found that there were no significant differences in the relationship between the degree of hyperandrogenism and the metabolic parameters associated with metabolic syndrome. The level or degree of hyperandrogenism did not alter the metabolic parameters in our study group.

 While it is known that PCOS is significantly more prevalent among obese patients [30] and altered metabolic parameters are directly associated to degrees of BMI [31], there is still no consensus as to the question of what is primary or secondary metabolic syndrome or hyperandrogenism. PCOS is polygenic condition and may be influenced by environmental factors like diet and lifestyle. In first-degree female relatives of PCOS patients, it has been documented to be an increase in adrenal androgens and insulin resistance in these girls thus suggesting a genetic component. However, to date, there is no clearly defined genetic etiology that is associated with PCOS and it is not clear whether hyperandrogenism is involved in the pathogenesis of this disorder.

We may speculate that increased rate of metabolic syndrome among obese girl with PCOS may be confounded with obesity rather than hyperandrogenism. Another reason for lack of correlation between PCOS and metabolic syndrome in adolescent girls may be that patients should be exposed to androgens longer to develop metabolic abnormalities in the adult age.

It is important for further studies to investigate the effects of prolonged exposure to high circulating androgens mainly in lean PCOS patients and the progression of this phenotype to metabolic syndrome. It is our belief that there may be an associated pathophysiological link with obesity, particularly visceral adiposity, and related adipose tissue factors, coagulation abnormalities, or insulin resistance [16, 19, 32] that may independently increase cardiovascular risk rather than a direct association of hyperandrogenism with metabolic syndrome morbidities.

Our study was limited by its small sample size. There can be limitations arising from the sample collection method or the chemiluminescent immunoassay method used to determine testosterone levels. However, this is unlikely to alter the main conclusions.

## Figures and Tables

**Table 1:** Distribution of main metabolic parameters between groups.

	PCOS		No PCOS	
	HT (*N* = 23)	IT (*N* = 31)	ONT (*N* = 38)	C (*N* = 15)
Age	16.6 ± 1.7	16.0 ± 1.4	15.9 ± 1.4	16.2 ± 1.9
Wt/Ht	33.4 ± 7.1	29.5 ± 6.5	33.3 ± 8	21.6 ± 2.1^∗#*∧*^
BMI	30 ± 7	29 ± 9.4	37 ± 9	24 ± 4^∗#*∧*^
SBP	120 ± 11	122 ± 12	123 ± 11.2	110 ± 9.4^∗#*∧*^
DBP	70.5 ± 8	73.5 ± 7.8	71 ± 5.2	65 ± 8
Glucose	81.6 ± 9	82.4 ± 11.3	92 ± 33	87 ± 7
HDL	50 ± 11	52.3 ± 14.3	46 ± 7.46	46 ± 9
TG	75 ± 39	74 ± 38.5	75 ± 32	75 ± 38
LH	16.2 ± 6.5	16.2 ± 6.5	10 ± 7.4	14 ± 12
Prolactin	11.1 ± 3.2	9.2 ± 4.7	9.2 ± 4.7	11 ± 4.2

*P < 0.05 between C and ONT, ^#^ *P* < 0.05 between C and IT, ^*∧*^ *P* < 0.05 between C and HT. HT: High Testosterone; IT: Intermediate Testosterone; ONT: Obese and Normal Testosterone; C: Control; Wt/Ht weight over height, BMI: Body mass index, SBP: systolic blood pressure, DBP: diastolic blood pressure, HDL: High-density lipoprotein, TG: triglycerides, LH: Luteinising hormone.

**Table 2:** Correlations of BMI with the studied parameters.

	*r*	*P* value
SBP	0.5	<0.001
DBP	0.23	<0.001
Total cholesterol	0.25	<0.01
HDL	0.27	<0.01
LDL	0.33	<0.01
LH	0.19	0.03
Triglycerides	0.06	0.56
FSH	0.06	0.49
Prolactin	0.08	0.35

BMI: Body mass index, SBP: systolic blood pressure, DBP: diastolic blood pressure, HDL: High-density lipoprotein, LDL: Low-density lipoprotein, LH: Luteinising hormone, FSH: Follicle-stimulating hormone.
